# Sink Drains in a Neonatal Intensive Care Unit: A Retrospective Risk Assessment and Evaluation

**DOI:** 10.3390/ijerph20176692

**Published:** 2023-08-31

**Authors:** Julia S. Schneider, Neele J. Froböse, Thorsten Kuczius, Vera Schwierzeck, Stefanie Kampmeier

**Affiliations:** 1 Institute of Hygiene, University Hospital Münster, 48149 Münster, Germany; julia.schneider@ukmuenster.de (J.S.S.); thorsten.kuczius@ukmuenster.de (T.K.); vera.schwierzeck@ukmuenster.de (V.S.); 2 Institute of Medical Microbiology, University Hospital Münster, 48149 Münster, Germany; neelejudith.froboese@ukmuenster.de; 3 Institute for Hygiene and Microbiology, University of Würzburg, 97080 Würzburg, Germany

**Keywords:** intensive care, neonatal, wastewater, *Pseudomonas*, *Klebsiella*, *Enterobacter*, transmission

## Abstract

Water systems in health care facilities can form reservoirs for Gram-negative bacteria. While planning a new neonatal intensive care unit (NICU), we performed a retrospective evaluation of potential risks from water-diverting systems on the existing NICU of our tertiary care University Hospital. During 2017 to 2023, we recorded nine nosocomial cluster events with bacterial pathogens in our NICU. Of these, three clusters of Gram-negative bacteria were potentially related to sink drains: A *Klebsiella oxytoca*, a *Pseudomonas aeruginosa*, and an *Enterobacter hormaechei* cluster were uncovered by clinical routine screening of patients and breastmilk samples. They were confirmed using whole-genome sequencing and a subsequent core genome multilocus sequence typing (cgMLST) algorithm. Our observations highlight that the implementation of sink drains in a NICU may have negative effects on patients’ safety. Construction planning should concentrate on the avoidance of washbasins in patient rooms when redesigning sensitive areas such as NICUs.

## 1. Introduction

Sink drains are well-known sources of nosocomial colonization and infection of patients with pathogenic bacteria in hospitals [1,2]. Transmission to patients can occur via droplets through water splashing back from sink drains, for example, while hand washing or via contact transmission due to contaminated hands or surfaces of patient surroundings. In particular, the transmission of multidrug-resistant Gram-negative bacteria (MDRGN) via sink drains in health care facilities is a recurring problem. Numerous outbreaks, for example of *Pseudomonas aeruginosa* (*P. aeruginosa*) [3,4,5], *Klebsiella* spp. [6,7], and *Enterobacter* spp. [8], have been described. In addition, the decontamination of affected sink drains can be difficult and is time-intensive due to the limited accessibility to mechanical cleaning and disinfection measures, as well as the high tenacity of pathogens in this area due to their ability to form biofilms [9,10,11,12]. 

Based on this, the current national German guidelines on hygiene requirements for wastewater-carrying systems recommend to avoid washbasins in patient rooms when planning new constructions [13]. This recommendation concerns medical areas where patients with an increased risk of infection are cared for or where increased antibiotic use is necessary, for example, neonatal intensive care units (NICUs) or hematopoietic stem cell transplantation wards. 

Because of these recommendations, when a new NICU in our tertiary-care hospital was planned, we performed a retrospective evaluation to determine the extent of the potential risks from sink drains in the existing NICU from 2017 to 2023. Thus, we sought to provide a basis for evaluating the structural measures that can minimize the risk of pathogen transmission from sink drains and reduce the incidence of infections from this source in the future.

## 2. Materials and Methods

### 2.1. Clinical Setting 

We examined the interdisciplinary NICU in the period of January 2017 to the end of February 2023. During this time period, 2149 patients were admitted to the NICU of the tertiary-care University Hospital Münster (UHM), comprising, in total, 18 beds. Temperature (19–20 °C) and humidity (50%) were kept at a constant level by the air conditioning system on the ward. The treated patient clientele of this NICU include premature infants, infectiological and postoperative cardiac surgery patients, and oncological and immunodeficient patients. 

### 2.2. Multidrug-Resistant Bacteria Screening and Infection Control Measures

In accordance with the national German guidelines, every patient in the NICU is routinely screened for multidrug-resistant bacteria (MDR), concentrating on methicillin-resistant *Staphylococcus aureus* (MRSA), vancomycin-resistant enterococci (VRE), and MDRGN, at least once per week [14,15,16]. For this purpose, microbiological swab samples are taken from the nasal vestibule, the pharynx, and the anal canal, as well as from individual possibly colonized/infected areas like wounds or a newborn’s umbilicus. Patients with a confirmed MDR status are isolated together with their parents in a separate room. Concomitantly, contact precaution measures are implemented to prevent the further transmission of MDR: ward staff and visitors are instructed to wear gowns; gloves; and, in the case of MRSA colonization, a mask during contact with the patient or the patient surroundings. To prevent transmission via surfaces, a routine treatment of all contact surfaces with the disinfectant IncidinTM plus 0.5% (ECOLAB Healthcare, Monheim am Rhein, Germany) is performed at least once a day.

### 2.3. Microbiological Sample Processing

For the detection of MRSA, swabs are plated onto selective solid culture media. The chromogenic agar plates (chromID™ MRSA Agar, bioMérieux, Marcy l’Étoile, France) are incubated under aerobic conditions at 36 ± 1 °C for 24 h. Suspect colonies are identified with Matrix-assisted Laser Desorption/Ionization Time of Flight Mass Spectrometry (MALDI-TOF MS) measurement using the Microflex instrument (Bruker, Bremen, Germany). If *S. aureus* complex (*S. aureus*, *S. argenteus*, *S. schweitzeri*, or *S. roterodami*) is detected, a PBP2a test (Clearview™ PBP2a SA Culture Colony Test, Abbott, Cologne, Germany) is performed, followed by resistance testing using a Vitek 2 automated system (bioMérieux, Marcy l’Étoile, France). 

For the detection of VRE, swabs are plated on selective plates (VRE Select™, Marnes-la-Coquette, France), which are incubated for 48 h at 36 ± 1 °C under aerobic conditions. The identification of suspicious colonies is carried out using MALDI-TOF MS. A disk diffusion test for phenotypic resistance testing is performed (Mueller Hinton II agar, BD, Heidelberg, Germany), and incubation takes place for 18–24 h at 36 + 1 °C under aerobic conditions. Zone diameters are assessed using the current European Committee on antimicrobial susceptibility testing (EUCAST) clinical breakpoints. Glycopeptide resistance is determined by molecular diagnostics (eazyplex^®^VRE, AmplexDiagnostics GmbH, Gars-Bahnhof, Germany) detecting *vanA* and *vanB* genes.

For detection of MDRGN (*Acinetobacter* spp., Enterobacterales and *P. aeruginosa*), four selective solid culture media are used (MacConkey II Agar, BD, Heidelberg, Germany; chromID^®^ ESBL Agar, bioMérieux; CHROMagar™ Acinetobacter, Mast Group, Reinfeld, Germany; Pseudomonas CFC agar, BD) and incubated for a maximum of 48 h at 36 °C under aerobic conditions. Suspected colonies are identified by MALDI-TOF MS and resistance testing is performed using a Vitek 2 automated system. If a reduced carbapenem susceptibility is detected in Enterobacterales or *Acinetobacter* spp., the finding is confirmed by phenotypic carbapenemase testing (MASTDISCS^®^Combi Carbaplus (Enterobacterales) D73C Test, Mast Group), carbapenemase PCR (eazyplex^®^ SuperBug CRE, AmplexDiagnostics GmbH), and repeated carbapenemase susceptibility testing using Etests (bioMérieux).

### 2.4. Surveillance of Breastmilk 

In order to uncover further sources of transmissions of pathogens early on, additionally, microbiological screening of every initial breastmilk sample is performed. Breastmilk, thereby, simultaneously serves as a surrogate parameter for maternal and patient colonization. 

For the detection of bacteria in breastmilk, 10 µL of each sample is plated on Columbia sheep blood agar (Oxoid, Wesel, Germany) and MacConkey II Agar as a selective agar for Gram-negative bacteria, then incubated for 48 h at 36 °C. Further differentiation of suspect colonies is performed using MALDI-TOF-MS. 

### 2.5. Environmental Surveillance and Infection Control Measures

According to the German Drinking Water Ordinance, regular fresh water samples are tested for the presence of *E. coli*, *Enterococci,* and *Pseudomonas aeruginosa,* as well as for the presence and quantity of *Legionella* spp. [17]. Samples are taken in order to check the water quality in the house installation according to the routine conditions for water analyses. Volumes of 100 mL are filtrated through 0.45 µm-membrane filters according to DIN EN ISO 19458, and the filters are placed on selective agar plates. *E. coli* and other coliform bacteria are identified on chromogenic coliform (CCA); *Pseudomonas aeruginosa* on cetrimide (CN); *Enterococci* on *Enterococci* agar plates; and *Legionella* on GVPC plates, respectively (Xebios, Düsseldorf, Germany), along with screening of species-specific colony colors and morphologies. In the case of irregularities in those tests, point-of use water filters are installed in front of the fresh water outlets as an immediate measure. Currently, there are no permanently installed disinfection measures, such as thermal or UV-disinfecting siphon systems, in the area of the NICU which was examined. 

Environmental sampling of hand contact and inanimate surfaces is performed specifically in cases of suspected transmission. As a precautionary measure against splashing water from sinks, splash guards are installed next to the washbasins to shield the surrounding area from the possible transmission of pathogens from the sink drains.

Environmental sampling is performed selectively by applying premoistened Polywipe™ (Medical Wire & Equipment, Corsham, UK) to hand contact surfaces, sink drains, or the immediate surroundings of sinks. The samples are incubated in Tryptic Soy Broth with Lecithin Tween and Histidin C LTHC (PharmaMedia, Leimen, Germany) for 48 h at 36 °C. A volume of 10 µL of each incubated sample is applied to Columbia sheep blood agar. Further differentiation of suspect colonies is performed using MALDI-TOF-MS.

### 2.6. Whole-Genome Sequencing-Based Typing 

For genetic comparison, in the case of epidemiologically detected clusters, bacterial isolates are subjected to WGS-based typing using either the MiSeq platform (Illumina Inc., San Diego, CA, USA) or the Sequel II platform (Pacific Biosciences Inc., Menlo Park, CA, USA). The Nextera XT protocol (Illumina Inc.) is used to prepare genomic DNA for MiSeq sequencing. After sequencing, quality trimming and de novo assembly using the SKESA algorithm coding regions are compared using a gene-by-gene approach, i.e., cgMLST, using the SeqSphere+ software version 8.3.1 (Ridom GmbH, Münster, Germany) as described previously [18,19]. 

For Pacific Biosciences sequencing, we constructed a sequence library using the SMRTbell Express Template Prep Kit 2.0 (Pacific Biosciences Inc.) in accordance with the manufacturer’s recommendations. The resulting reads were then assembled by applying the “Microbial Assembly” pipeline within the SMRT Link software version 9 (Pacific Biosciences Inc.), using the default parameters.

For cgMLST analyses, the public cgMLST scheme for *P. aeruginosa* [20], as well as unpublished in-house schemes for the *E. cloacae* (reference genome: ATCC 13047) and *K. oxytoca* complexes (reference genome: KCTC 1683) were applied. Key antimicrobial resistance genes were determined using the NCBI AMRFinderPlus integrated in the SeqSphere+ software [21]. The clonal relationships between genotypes were displayed using a minimum spanning tree (MST) algorithm, calculated using the same software. 

### 2.7. Ethics Statement

All strategies and investigations were performed in accordance with the national recommendations for outbreak investigations of the legally assigned German institute for infection control and prevention (Robert-Koch Institute). Formal consent was, therefore, not required.

## 3. Results

**Figure 1:**
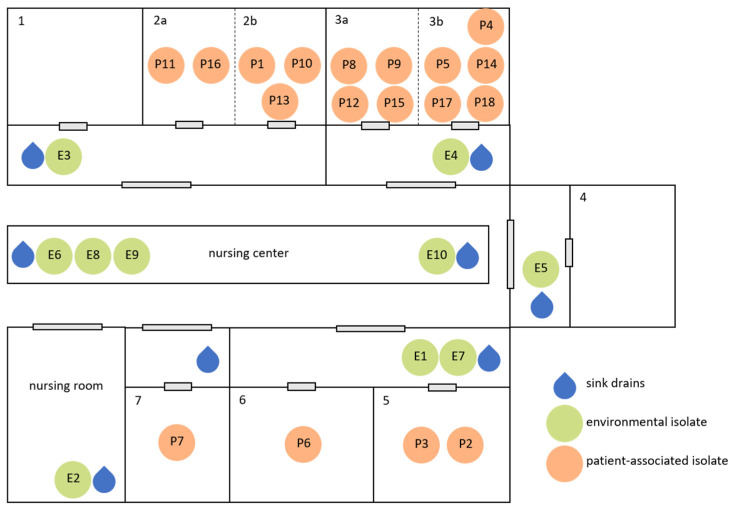
Distribution of sink drains/wash basins in the investigated NICU, as well as environmental (E, green) and patient-associated (P, orange) isolate locations during the three cluster events, listed in chronological order (Table 1, Table 2 and Table 3).

During the observation period, nine cluster events with bacterial pathogens were identified in the NICU. Of these, three were potentially related to sink drains (see also Figure 1).

### 3.1. Environmental, Patient, and Breastmilk Testing 

From June to July 2017, *Klebsiella oxytoca* (*K. oxytoca*) was isolated from six different patients during the routine colonization screenings. This observation was investigated in August 2017 by means of extensive environmental testing. In this investigation, *K. oxytoca* was isolated from environmental testing samples of washbasins in two separate rooms. 

From October 2021 to January 2022, *P. aeruginosa* was isolated from five different patients during routine screening, and two breastmilk samples were utilized, which originated from two different mothers of patients that were colonized with *P. aeruginosa*. Subsequently, environmental testing of the hand contact surfaces and washbasins in November 2021 revealed *P. aeruginosa* contaminations in five samples, which were collected from four different sinks in separate rooms. 

In January 2023, an *Enterobacter cloacae* complex was found in the microbiological routine tests of eight different patients. Here, environmental testing uncovered *Enterobacter hormaechei* (*E. hormaechei*) in three environmental samples from two different sink drains in one room. 

### 3.2. Whole-Genome Sequencing-Based Typing

Eight *K. oxytoca*, nine *P. aeruginosa,* and eleven *E. hormaechei* isolates were subjected to WGS. 

**Figure 2:**
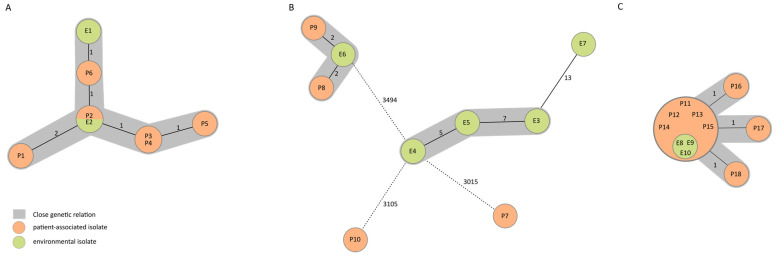
Minimum spanning trees of *K. oxytoca* (**A**), *P. aeruginosa* (**B**), and *E. hormaechei* (**C**) isolates, numbered in chronological order.

All of the *K. oxytoca* isolates, from both the patient and washbasin samples, formed genetic clusters with either identical or closely related strains that showed, at maximum, one to four differing alleles using the cgMLST algorithm (Figure 2A). All isolates were resistant to ampicillin and cefaclor based on phenotypic susceptibility testing. In the WGS analyses, all isolates showed the presence of ß-lactamase genes (*bla*_OXY-2-1_).

The WGS of *P. aeruginosa* strains resulted in two clusters. One distantly related environmental and two genetically unrelated patient isolate singletons were found: Cluster 1 comprised patient and environmental isolates, while cluster 2 consisted only of environmental isolates (Figure 2B). No isolates showed antibiotic resistances according to phenotypic susceptibility testing. Based on WGS data, the isolates were identified as ST 309 and carried antibiotic-resistant ß-lactamase genes (*bla*_OXA_, *bla*_PDC-19a_).

All patient and environmental *E. hormaechei* isolates constituted a genetic cluster with identical and closely related strains, with a maximum distance of one allele in this cluster (Figure 2C). All isolates showed phenotypic resistance to amoxicillin, ampicillin/sulbactam, and cefpodoxim. Three isolates showed phenotypic resistance to piperacillin and to third-generation cephalosporins. One isolate showed phenotypic resistance to ertapenem. In the WGS analysis, all isolates showed the presence of a β-lactamase gene (*bla*_ACT-17_).

No water quality irregularities were detected in regular fresh water sampling from 2017 to 2023. In particular, no *K. oxytoca*, *P. aeruginosa*, or *E. hormaechei* was detected. 

Detailed information on the origin of the samples which were subjected to WGS, their respective localizations, and isolation data are given in Figure 1 and Table 1, Table 2 and Table 3.

Minimum spanning trees of seven environmental (green) and ten patient-associated (patient and breast milk, orange) isolates display the genotypic relationships based on the 4638 (A) 3867 (B), and 2626 (C) target genes, respectively, and pairwise ignoring missing values. Each circle represents one genotype, and the numbers next to the connecting lines show the number of allelic differences between genotypes. Grey coloring indicates a close genetic relation.

## 4. Discussion

The establishment of water-carrying systems in NICUs is still controversially discussed. On the one hand, nursing staff have become accustomed to having direct access to water for patient care. On the other hand, water-supplying systems are potential sources of hospital-acquired pathogens. In order to determine the true possibility of transmission of these pathogens, we, therefore, analyzed nosocomial clusters of pathogens associated with water-carrying systems both epidemiologically and by means of molecular typing.

Over a period of seven years, three transmission events with either *P. aeruginosa*, *K. oxytoca*, or *E. hormaechei* became evident on our NICU, involving both colonized patients and contaminated environmental surfaces, including washbasins and sink drains. This observation is particularly relevant as neonatal patients are at enhanced risk for hospital-acquired infections with these colonizing pathogens [22,23]. This is attributed to patient-associated risk factors as low birth weight, immaturity of the immune system, and a lack of gut barrier integrity [24,25]. Additionally, Gram-negative pathogens especially are associated with water-supplying systems, bearing an increased potential to develop antibiotic multi-resistance, which is critical as there are limited anti-infective therapeutic options in pediatric patients. 

As systematic data on this issue are scarce, herein, we analyzed NICU cluster events using molecular typing methods. The cgMLST analysis which was performed showed a close genetic relationship between the detected patient and environmental isolates. Thereby, pathogen transmission from patient to sink drains or vice versa also appeared if the sink drain was not located in the same room as the patient. Thus, two modes of transmission in these scenarios are conceivable: (i) Transmission from sink drains to patients can occur through droplets or splashes of contaminated water [26]; or (ii) water-associated pathogens spread via (in)direct contact and insufficient hand or surface disinfection. The latter, in particular, emphasizes the need for intensified cleaning and disinfection of patient surroundings in addition to proper hand hygiene, including continuous training and compliance observations of the responsible personnel. 

Regardless of the direction of contamination, sink drains form a reservoir for Gram-negative pathogens along the wastewater pipes that must be controlled [27]. Hence, there are multiple attempts to prevent pathogen transmission from sink drains to patients. Avoiding the use of washbasins in patient rooms is one possibility, as recommended by the German national guidelines [13]. Additionally, the implementation of waterless patient care is an option [5]. Disinfection of sink drains, either chemically or thermally, to eradicate the pathogens can be attempted, but is often difficult and not always successful because of the ability of pathogens to form biofilms and persist over a long time in humid environments like sink drains [4,28]. Alternatively, mechanical barriers can be an effective solution to reduce splashing and contamination of the patient environment. Thus, these have already been established on stem cell transplantation wards [4]. If the implementation of water-supplying systems in patient rooms is essential, intensified environmental screening strategies to uncover the sources of critical pathogens come into play. These can be established in different manners, e.g., using a weekly routine or after a room is occupied by a patient who is colonized/infected with a relevant pathogen. Prospective controlled studies on NICUs are thus needed in order to verify the effectiveness of such implementations. 

Our study has limitations. We present a monocentric study, which is of retrospective nature. Hence, the data interpretation cannot be generalized, and additional sample collection during the investigation period was not possible for comparison purposes. However, in the literature, several outbreak events in hospitals involving MDRGN of these species associated with sink drains can be found [7,26]. Therefore, our observation represents part of the main spectrum of the Gram-negative bacteria in hospital sink drains. In addition, we compared the present isolates with water-associated in-house isolates originating from wards other than the NICU (e.g., stem cell transplantation unit) and could not detect a close genetic relationship, implying that this is not ward-specific, but a general concern. 

## 5. Conclusions

Sink drains represent a relevant source of pathogen transmission on hospital wards. We suggest that the national recommendation should contain a risk-adapted categorization for the establishment of sink drains in newly built patient rooms. Based on the data shown herein, on existing NICU wards, water supply units should be reduced to a minimum, while intensified hand hygiene and surface disinfection should be promoted in order to reduce pathogen transmission.

## Figures and Tables

**Table 1:** *K. oxytoca* originating from screening and environmental samples.

Isolate no.	Isolation Date	Localization
P1	18/06/2017	Throat swab
P2	25/06/2017	Anal swab
P3	09/07/2017	Anal swab
P4	17/07/2017	Throat swab
P5	17/07/2017	Anal swab
P6	24/07/2017	Anal swab
E1	15/08/2017	Washbasin
E2	15/08/2017	Washbasin

**Table 2:** *P. aeruginosa* originating from screening, breast milk, and environmental samples.

Isolate no.	Isolation Date	Localization
P7	28/10/2021	Throat swab
E3	29/10/2021	Washbasin
E4	29/10/2021	Washbasin
E5	29/10/2021	Washbasin
E6	29/10/2021	Washbasin 1
E7	29/10/2021	Washbasin
BM1 (P8)	06/11/2021	Breast milk
BM2 (P9)	07/11/2021	Breast milk
P10	17/01/2022	Throat swab

**Table 3:** *E. hormaechei* originating from screening and environmental samples.

Isolate no.	Isolation Date	Localization
P11	23/01/2023	Anal swab
P12	23/01/2023	Anal swab
P13	23/01/2023	Throat swab
P14	31/01/2023	Anal swab
P15	31/01/2023	Anal swab
P16	31/01/2023	Anal swab
P17	31/01/2023	Throat swab
P18	02/02/2023	Anal swab
E8	01/02/2023	Washbasin 1
E9	01/02/2023	Washbasin 1
E10	01/02/2023	Washbasin 2

## Data Availability

The data presented in this study are available upon request from the corresponding author.
